# Editorial Note: Determinants of neonatal near misses among neonates admitted to Guji and Borena zones selected public hospitals, Southern Ethiopia, 2021: A facility based unmatched case control study design

**DOI:** 10.1371/journal.pgph.0004850

**Published:** 2025-06-24

**Authors:** The PLOS Global Public Health Editors

This article [1] was identified as one of a series of articles for which the *PLOS Global Public Health* Editors have concerns about authorship and adherence to the journal’s first and second publication criteria. We regret that the issues were not addressed prior to the article’s publication.

In addition, the article’s Data Availability statement was incorrect and is updated to: Data essential for the conclusion are included in this manuscript. Additional data can be obtained from the corresponding author upon reasonable request.
